# Differential Expression of lncRNA-miRNA-mRNA and Their Related Functional Networks in New-Onset Type 2 Diabetes Mellitus among Chinese Rural Adults

**DOI:** 10.3390/genes13112073

**Published:** 2022-11-09

**Authors:** Yu Song, Luting Nie, Mian Wang, Wei Liao, Changsheng Huan, Zexin Jia, Dandan Wei, Pengling Liu, Keliang Fan, Zhenxing Mao, Chongjian Wang, Wenqian Huo

**Affiliations:** 1 Department of Occupational and Environmental Health, College of Public Health, Zhengzhou University, Zhengzhou 450001, China; 2 Department of Epidemiology and Biostatistics, College of Public Health, Zhengzhou University, Zhengzhou 450001, China

**Keywords:** long noncoding RNA, microRNA, messenger RNA, new-onset type 2 diabetes mellitus

## Abstract

Increasing evidence suggested that the expression and inter-regulation of long noncoding RNA (lncRNA), microRNA (miRNA), and messenger RNA (mRNA) were related to the development of diabetes. Based on bioinformatics analysis, this study aimed to comprehensively analyze the dysregulated RNA molecules related to new-onset type 2 diabetes mellitus (T2DM). Twenty-four patients with new-onset T2DM were included as cases, and sex- and age-matched participants were included as controls. The differentially expressed lncRNAs, miRNAs, and mRNAs between the two groups were screened by RNA sequencing. LncRNA-miRNA-mRNA network and enrichment analysis were used to reveal the RNA molecules that were potentially associated with T2DM and their early changes. A total of 123 lncRNAs, 49 miRNAs, and 312 mRNAs were differentially expressed in the new-onset T2DM (fold change ≥ 1.5 and *p* value < 0.05). Functional analysis revealed that differentially expressed RNAs were likely to play essential roles in diabetes-related pathways. In addition, the protein–protein interaction (PPI) network screened multiple hub mRNAs, and lncRNA-miRNA-mRNA networks showed that a single miRNA could be related to multiple lncRNAs, and then they coregulated more mRNAs. SLC25A4, PLCB1, AGTR2, PRKN, and SCD5 were shown to be important mRNAs in T2DM, and miR-199b-5p, miR-202-5p, miR-548o-3p as well as miR-1255b-5p could be involved in their regulation. In conclusion, several new and previously identified dysregulated lncRNAs, miRNAs, and mRNAs were found to be vital biomarkers in T2DM. Their alterations and interactions could modulate the pathophysiology of T2DM. Those findings may provide new insights into the molecular mechanisms underlying the development of T2DM.

## 1. Introduction

Diabetes, a metabolic disease, is one of the most common chronic diseases with high morbidity and mortality rates in the population. According to the International Diabetes Federation (IDF) Diabetes Atlas, there were 573 million adults with diabetes worldwide in 2021, and this figure was expected to increase to 783 million by 2045 [1]. In China, about 113.9 million and 493.4 million adults had diabetes and pre-diabetes, respectively [2]. Notably, research showed that the prevalence of diabetes increased more rapidly in the rural population than in the urban population [3]. Furthermore, type 2 diabetes mellitus (T2DM) accounts for about 90% of people with diabetes, and most patients have multiple complications that seriously threaten human health [4,5]. Epidemiological evidence suggested that changes in diet habits and lifestyle had been considered risk factors for T2DM [6]. Furthermore, it is increasingly recognized that genetics contribute to T2DM [7].

Noncoding RNA (ncRNA) has protein-coding regions but cannot be translated into protein for the most part. Long noncoding RNA (lncRNA) and microRNA (miRNA), the two major members of the ncRNA family, have been documented to exert vital roles in the regulation of the pathophysiological processes of T2DM [8,9]. MiRNA, a noncoding single-stranded RNA molecule of approximately 22 nucleotides in length, can bind to the 3′-untranslated region (3′-UTR) of messenger RNA (mRNA) through imperfect complementarity to perform post-transcriptional regulation of gene expression [10]. LncRNA is more than 200 nucleotides in length, which contribute to various life activities such as epigenetic regulation, cell cycle regulation, and cell differentiation regulation. In recent years, lncRNA has become a genetics hotspot. LncRNAs and mRNAs are competing endogenous RNAs (ceRNAs), and their expressions were positively correlated. MiRNAs can silence genes by binding corresponding mRNAs, while ceRNAs can effectively control post-transcriptional regulation by competitively binding the same corresponding miRNA response element (MRE) [11]. 

Early detection is essential for the treatment of diabetes as well as the prevention of its complications. Existing validated traditional markers have been used in diagnosing diabetes and its complications, but there are still some limitations to using these markers. For example, dietary factors and duration of fasting had a significant effect on blood glucose fluctuations. Furthermore, some biomarkers, such as TNF-α, PON-1, IL-6, and insulin-like growth factor 2, could reflect and help predict the development of diabetes to some extent but are not yet adequate for clinical application. Recent research showed that ncRNAs, previously thought of as “noise”, became a new direction in predicting a wide range of diseases, and differentially expressed ncRNA could be used as a predictor of diseases. Of note, previous studies showed that ncRNAs were linked to the development of T2DM, insulin secretion, islet cells, and endothelial endoplasmic reticulum stress [12,13]. Specifically, in 2004, miR-375 was first reported to regulate murine islet secretion directly. MiR-375 can reduce insulin secretion by directly targeting 3′-phosphatidylinositol-dependent kinase 1 mRNA, which was a critical molecule in the intracellular phosphatidylinositol kinase (PI3K) pathway [14]. A recent study in Egyptian children found that miR-25 exerted antioxidant and anti-apoptotic effects through activating the PTEN/Akt pathway and was downregulated in diabetics [15]. In addition, lncRNA- p3134 was reported to have an important role in insulin signaling and glucose metabolism in pancreatic β cell in adults from Guangzhou, China [16]. With the advancement of diabetes research, the lncRNA-miRNA-mRNA network has been found to play a pivotal role in regulating glucose homeostasis [17,18]. Studies suggested that lncRNA-H19/let-7 negative feedback was associated with glucose metabolism in muscle cells. LncRNA-H19 could combine with let-7 to affect the expression of let-7 target genes, including insulin receptor gene and lipoprotein lipase gene, ultimately blocking insulin signaling, and reducing glucose intake [19]. 

As noted above, lncRNA-miRNA-mRNA inter-regulation contributed to the development of T2DM and its related risk factors. However, there were fewer reports on RNA-mediated regulatory networks in patients with new-onset T2DM, especially in rural Chinese populations, and many unknown RNA molecules remain to be explored. Therefore, this study intended to comprehensively analyze the key RNA molecules related to T2DM in lncRNA-miRNA-mRNA networks based on bioinformatics analysis and explore the possible biological processes and signaling pathways involved.

## 2. Materials and Methods

### 2.1. Participants

Participants in this study were drawn from a prospective cohort of Chinese rural adults in Henan Province. (Registration Number: ChiCTR-OOC-15006699) [20]. After excluding type 1 diabetes and other causes of diabetes, 24 new-onset T2DM cases aged 41 to 69 years were randomly selected from four townships of Xuchang County in 2020, as well as 24 healthy controls. Controls were selected according to the sex and age of the relevant T2DM cases. There were six T2DM patients or healthy controls in each mixed sample. Finally, four mixed samples of T2DM and four sex-and age-matched normal glucose tolerance (NGT) controls were obtained. According to the diagnostic criteria of the American Diabetes Association (ADA) (2002) and the WHO (1999), the oral glucose tolerance test was performed on participants without diabetes, and then a combination of their blood glucose at the four test time points, plus clinician experience, was used to determine whether they were new-diagnosed T2DM patient.

### 2.2. Ethical Approval and Informed Consent

The study was in accordance with the 1975 Declaration of Helsinki and was supported by the Ethics Committee of Zhengzhou University Life Science (Code: [2015] MEC (S128)). All participants completed written informed consent.

### 2.3. Data Collection and Laboratory Measurements

Trained investigators used a constructed questionnaire to get information on socio-demographic characteristics. This study also collected anthropometric data such as height, weight, and blood pressure levels. BMI was calculated as weight in kilograms divided by the square of height in square meters. Blood pressure measurement was repeated three times by using a sphygmomanometer (HEM-7071A). The average value of systolic/diastolic blood pressure (SBP/DBP) was analyzed. Pulse pressure (PP) was calculated by SBP minus DBP. In addition, an updated homeostasis model (HOMA) was used to estimate the insulin resistance (IR), and defined as HOMA-IR. Venous blood samples were collected after an overnight fast for at least 8 h. Plasma and serum samples were separated from whole blood by centrifugation at a relative centrifugal force of 2000× *g* for 10 min at room temperature. Serum insulin (INS) and fasting blood glucose (FBG) were measured by radioimmunoassay and Cobasc501 automatic biochemical analyzer, respectively.

### 2.4. RNA Isolation and Sequencing

Total RNA was extracted from plasma using the Trizol Kit (Life Technologies, Carlsbad, CA, USA). The total RNA purity and integrity were assessed by using the NanoDrop 2000 (Thermo Fisher, Waltham, MA, USA) and the RNA Nano 6000 Assay Kit of the Agilent Bioanalyzer 2100 system (Agilent Technologies, Carpinteria, CA, USA).

Two sequencing libraries were constructed to study the expression profiles and general characteristics of the differentially expressed noncoding RNA (DEncRNAs) and differentially expressed mRNAs (DEmRNAs) in the human plasma sample. For lncRNA, the ribosomal RNA (rRNA) was further digested from the total RNA using Ribo-Zero Gold rRNA Removal Kit (MRZG12324, illumina). After removing rRNA, lncRNA and mRNA were purified suing Agencourt RNA Clean XP Beads. The purified RNA samples were cut into short fragments, which were used as a template to synthesize the first and second strands of cDNA. The cDNA fragment is ligated to the Illumina sequencing adapter after end repair and addition of poly (A). The ligation products were enriched by PCR amplification to establish library for lncRNA and mRNA. Finally, the products were sequenced by using Illumina Novaseq 6000 by Gene Denovo Biotechnology Co. Ltd. (Guangzhou, China).

For miRNA, the 18–30 nt fragments were selected by agarose gel electrophoresis. After the addition of 5′ and 3′ adaptor ligations, the miRNA was reverse transcribed into cDNA and enriched by PCR amplification. Subsequently, the miRNA library was constructed after recovery and purification of bands by agarose gel electrophoresis. Finally, Illumina Novaseq 6000 was used for miRNA sequencing by Gene Denovo Biotechnology Co. Ltd. (Guangzhou, China). All raw transcriptome data are accessible through NCBI's Sequence Read Archive (SRA), under accession number PRJNA898658.

### 2.5. Quality Control of Raw Sequencing Data

For LncRNA and mRNA, first, clean reads were obtained by removing reads containing an ploy-N or adapter, and low-quality reads from raw reads. Subsequently, clean reads were compared to the ribosome database using bowtie2 [21]. In this step, the unmapped reads were maintained for subsequent transcriptome analysis. In addition, paired-end clean reads were aligned to the reference genome using HISAT2 software. For miRNAs, clean reads were retained by removing reads containing with 5′adapter contaminants, ploy-A, without 3′adapter, the insert tag or the insert fragment less than 18 nt in length, as well as low-quality reads. Furthermore, we performed standard data analysis. In this step, tags sequences were compared to the GenBank, reference genome, and Rfam using the software blastall 2.2.25 (blastn) and bowtie (v 1.1.2).

### 2.6. RNA Sequencing Data Analysis

FPKM values were calculated for each transcript using RSEM software. Differentially expressed lncRNA, miRNA, and mRNA between NGT and T2DM groups were identified by the edgeR package, and the fold change ≥1.5 and *p* value < 0.05 were considered statistically significant [22]. Moreover, reproducibility of samples in groups was assessed by performing sample gene expression correlation analysis. Volcano plots, heat maps, and radar plots were performed using ggplot2 and heat maps to visualize differential RNA expression profiles. We explored the possible T2DM-related differentially expressed RNAs (DERNAs) based on previous studies and our sequencing results combined with enrichment analysis. In addition, the correlations of DERNAs with HOMA-IR were analyzed using Spearman correlation analysis.

Statistical description was described as mean with standard deviation (SD) for continuous variables. Database analysis was conducted using SPSS software, version 21.0 (SPSS Inc., Chicago, IL, USA). 

### 2.7. Functional Enrichment Analysis

To better explore the mechanisms associated with the DERNAs of T2DM, Gene Ontology (GO) and Kyoto Encyclopedia of Genes and Genomes (KEGG) enrichment analyses were performed to predict the potential signal transduction pathways, biological functions of all DERNAs, and biochemical metabolic pathways. Among them, GO analysis has three ontologies, including biological process (BP), cellular component (CC), and molecular function (MF), through which DERNAs can be annotated for GO functional classification and analyzed for functional significance enrichment. Furthermore, GO term network was constructed using ClueGO, the functional plug-in for Cytoscape (v 3.7.1), to further explore the inter-regulatory functional mechanisms and screen out key RNA molecules.

A hypergeometric test with a threshold corrected-*p* value ≤ 0.05 was defined as significantly enriched GO terms while *Q* values ≤ 0.05 was defined as significantly differentially enriched pathways in the KEGG pathway analysis.

### 2.8. PPI Network Construction

The PPI network of DEmRNAs was constructed by STRING (https://string-db.org (accessed on 7 November 2021)). Those interactions with a validation score >0.4 were defined as significant, and the screened networks were visualized by Cytoscape (v3.7.1), and the functional enrichment were visualized and compared [23]. To further expose the hub molecules, the PPI network function module was established by molecular complex detection algorithm (MCODE), with degree cutoff = 2, K-core = 2, max. depth = 100, and node score cutoff = 0.2. It was a clustering algorithm identifying locally densely connected regions in PPI network based on node-weighting arithmetic.

### 2.9. LncRNA-miRNA-mRNA Network Construction

The lncRNA-miRNA-mRNA network was constructed based on the theory that ceRNAs bind miRNAs competitively through the same MREs. First, miRcode was used for the prediction of the potential lncRNA-miRNA pairs. Second, the miRNA-mRNA pairs were predicted using TargetScan (v7.0) and Miranda (v3.3a), and the intersection was used as the final result of miRNA target prediction. Based on the above results, the lncRNA-miRNA-mRNA pairs with *p*-values < 0.05 were selected as the final ones using the hypergeometric cumulative distribution function test.

Finally, the lncRNA-miRNA-mRNA network was structured by combining all the co-expressed competitive triplets identified above and visualizing them using Cytoscape software (v3.7.1).

## 3. Results

### 3.1. Characteristics of Study Characteristics

The basic characteristics of the participants are shown in Table 1. The median (IQR) of FBG in NGT and T2DM groups was 5.31 (0.51) and 8.25 (1.56) mmol/L, respectively. The median (IQR) of INS was 7.79 (5.02) versus 12.53 (5.04) mIU/L in the NGT and T2DM, respectively. However, there were no statistical differences in sex, BMI, SBP, and DBP among NGT and T2DM. In addition, the characteristics of the eight mixed samples are shown in Table S1.

### 3.2. Differential Expression of lncRNA, miRNA, and mRNA

**Figure 1:**
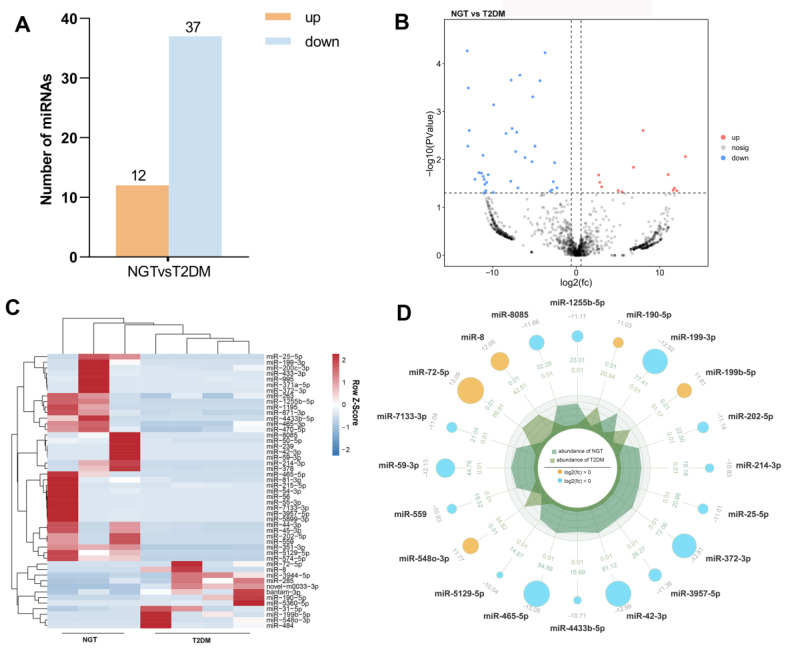
The expression profiles of miRNAs. (**A**) The column chart of the number of statistical DEmiRNAs; (**B**) the volcano plot displays the distribution of DEmiRNAs; (**C**) hierarchical clustering analysis of DEmiRNAs between T2DM group and NGT group were showed by the heat map. Red color represents up-regulated miRNAs, and the blue color represents down-regulated miRNAs; (**D**) the radar plots display the distribution of DEmiRNAs and the size of the circle indicates the log2 (FC). DEmiRNAs, differentially expressed microRNA; NGT, normal glucose tolerance; Log2 (FC), log2 (fold change); T2DM, Type 2 Diabetes.

Gene expression correlation analysis of the samples showed that the control 4 sample was the outlier and was excluded from the subsequent analysis (Figure S1). Differentially expressed lncRNA, miRNA, and mRNA between groups were recognized by the edgeR with the *p* value < 0.05 and fold change ≥ 1.5. The results showed that miRNA, lncRNA, and mRNA were significantly differentially expressed between the T2DM and NGT groups. As shown in Figure 1A, Figures S2A and S3A, a total of 49 miRNAs (12 up-regulated and 37 down-regulated), 123 lncRNAs (62 up-regulated and 61 down-regulated), and 312 mRNAs (146 upregulated and 166 downregulated) were differentially expressed in the T2DM group. The results showed that there were clear differences in the expression profiles of lncRNAs, miRNAs, and mRNAs through the volcano plots, heat maps, and radar plots. According to fold change and *p* value, the top 50 and top 20 DERNAs were presented in heat maps and radar plots, respectively (Figure 1B–D, Figures S2B–D and S3B–D).

According to previous studies and our sequencing results combined with enrichment analysis, 12 differentially significant miRNAs, mRNAs, and lncRNAs that may be related to T2DM were presented in Figures S4–S6 and more information was given in Tables S2–S4. Enrichment analysis of miRNA target genes revealed that miR-199b-5p was enriched in lipid metabolism, while miR-484 and miR-3944-5p were enriched in glucose and insulin regulatory regulation. In addition, mRNA including SLC25A4, SLC25A6, ATP6V1C2, and GOT1 were involved in regulating insulin secretion. GNPDA2, PRELID2, MBD5, GOT1, and SLC2A3 were enriched in glucose homeostasis or lipid metabolism. The results of correlation analysis suggested that partial DERNAs could be associated with IR (Tables S5–S7).

### 3.3. GO Functional and KEGG Pathway Analysis

**Figure 2:**
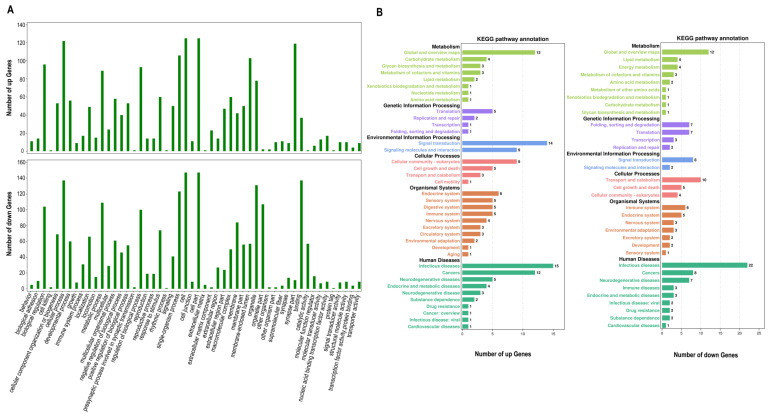
GO and KEGG pathway annotation of all DEGs. (**A**) GO annotation and classification of DEGs; (**B**) KEGG pathways in which DEGs were enriched (compared to the entire genome background). DEGs, differentially expressed genes. GO, gene ontology; KEGG, Kyoto Encyclopedia of Genes and Genomes.

GO and KEGG pathway analyses were performed to better explore the regulatory mechanisms of mRNA molecules in T2DM. GO ontologies include 52 subcategories, of which the enriched items included regulation of the biological process, metabolic process, cellular components, and molecular binding (Figure 2A). Moreover, KEGG pathway analysis suggested that the differential mRNA enrichment pathway was focused on the metabolism (including carbohydrate metabolism, glycan biosynthesis, lipid metabolism, and metabolism of cofactors as well as vitamins), genetic information processing (including translation and replication, repair and signal transduction, signaling molecules and interaction), human disease (including infectious disease, cancers, endocrine and metabolic diseases, transport and catabolism, immune system, and neurodegenerative diseases) (Figure 2B)).

**Figure 3:**
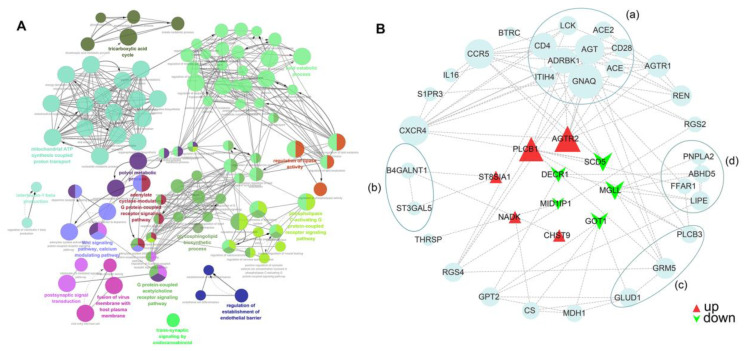
Biological function analysis of DEGs. (**A**) GO term network of DEGs. Each node stands for an enriched GO term (adjusted *p* value (Corrected with Bonferroni downgrading procedure) < 0.05). Nodes are interconnected when the gene overlap is >50% according to the kappa score; (**B**) protein–protein interaction (PPI) network. The four circle areas (**a**–**d**) were most significant cluster in MCODE analysis. DEGs, differentially expressed genes; GO, gene ontology; MCODE, molecular complex detection. More details are given in Tables S8–S10.

In the GO term network, a total of 15 GO functional groups were classified (Figure 3A and Table S8). Groups 5, 14, and 15 were associated with diabetes. The primary regulatory functions involved include fatty acid *β*-oxidation lipid storage, regulation of lipase activity, regulation of phospholipase activity, gluconeogenesis, as well as positive regulation of carbohydrate metabolic process, and main diabetes-related signaling pathways include phospholipase C-activating G protein-coupled receptor signaling pathway, Wnt signaling pathway, and glycosphingolipid biosynthetic process. The mainly involved differential mRNAs included AGTR2, PRNK, CYP19A1, MID1IP1, MGLL, and ST8SIA1 (Table S9).

### 3.4. The Protein–Protein Interaction (PPI) Network

To further investigated the function of DEmRNAs at the protein level, the PPI network for DEmRNA was constructed using STRING (https://string-db.org (accessed on 7 November 2021), including 41 nodes and 102 edges. The highest connectivity degrees were GNAQ (guanine nucleotide-binding protein G (q), degree = 14), AGT (angiotensinogen; essential component of the renin-angiotensin system, degree = 11), CCR5 (C-C chemokine receptor type 5, degree = 11), and CXCR4 (C-X-C chemokine receptor type 4, degree = 11). The top 20 core nodes and their corresponding degree are shown in Table S10. Furthermore, the modules of the PPI network were screened using MCODE, as shown in Figure 3B. We screened the top 4 clusters with the highest cluster scores and the main biological processes (Table 2). DEmRNAs, including AGTR2 and SCD5, were found to be involved in the regulations of renin-angiotensin production of blood volume and cytokines in diabetic cardiomyopathies, ST8SIA1, engaged in the metabolism of the Ganglio sphingolipids, GOT1, involved in carbohydrates, and PLCB1 as well as MGLL, participates in the regulation of lipolysis and insulin secretion as well as hormone levels by GPR40-binding fatty acids (FFAR1).

### 3.5. LncRNA-miRNA-mRNA Regulatory Network

**Figure 4:**
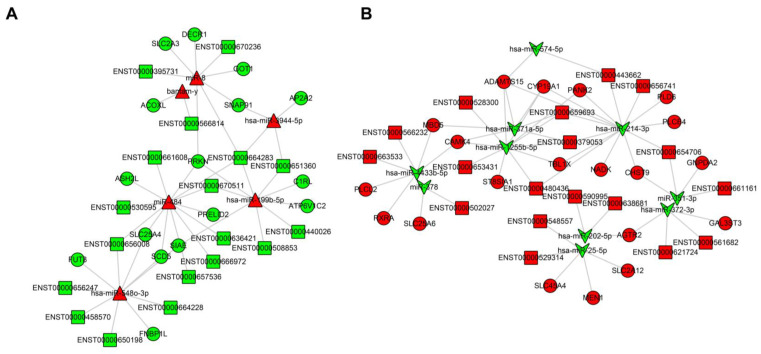
Metabolism-associated lncRNA–miRNA–mRNA interaction network for T2DM. (**A**) Red triangles represent up-regulated miRNAs, green squares represent down-regulated lncRNAs, and green circles represent down-regulated mRNAs; (**B**) green concave quadrilateral represent down-regulated miRNAs, red squares represent up-regulated lncRNAs, and red circles represent up-regulated mRNAs. The lncRNA-miRNA-mRNA regulatory network base on the DEGs in T2DM and was screened by correlation analysis and hypergeometric distribution tests. T2DM, Type 2 Diabetes; MRNA, messenger RNA; LncRNA, long noncoding RNA; MiRNA, microRNA.

The interaction network map for T2DM-related lncRNA-miRNA-mRNA is shown in Figure 4. There are 36 lncRNAs, 17 miRNAs, and 36 mRNAs in the lncRNA-miRNA-mRNA network. The ceRNAs network showed the possible interactions between lncRNA, miRNA, and mRNA. For example, PRKN was predicted to interact with miR-199b-5p and miR-484. LncRNAs ENST00000670511 (AC090825.1) and ENST00000664283 (STX18-AS1) were predicted to act as ceRNAs and compete for binding to miR-199b-5p and miR-484, thereby regulating the expression of PRKN. The up-regulated miR-484 and down-regulated lncRNA ENST00000666972 (MEG8) could down-regulate the expression of SCD5 (Figure 4A). Similarly, miR-1255b-5p could target both the gene ST8SIA1 and lncRNA ENST00000653431 (AL021368.5) (Figure 4B). RNAs connectivity in the lncRNA-miRNA-mRNA network was defined as the number of co-expressed targeted miRNAs. More details are shown in Tables S4 and S11.

## 4. Discussion

T2DM is a hyperglycemic metabolic disease caused by a variety of factors, which seriously threatens human health [24]. Due to the complexity of the mechanisms of metabolic diseases, the causes and pathogenesis of these diseases are not completely well understood, nor effective cures are available [25]. With the development of genomics and high-throughput sequencing technology, accumulating evidence indicated that RNAs were important regulators of the human body life activities and disease development, and their abnormal expression as well as mutations may be tightly linked to diabetes [26,27,28,29].

In the current study, there were clear differences in the expression profiles of lncRNAs, miRNAs, and mRNAs through the volcano plots, heat maps, and radar plots. A total of 123 lncRNAs, 49 miRNAs, and 312 mRNAs were differentially expressed in the T2DM group compared to the NGT. Among them, miR-199b-5p expression was upregulated in the T2DM group, while miR-25-5p and miR200c-3p were downregulated. In addition, functional analysis of DEmiRNA target genes also revealed that miR-25-5p, miR-199b-5p, and miR-200c-3p were involved in lipid and glucose metabolic processes. Those findings were consistent with previous studies that down-regulated miR-199b-5p expression and prevented diabetic nephropathy (DN) tubular injury [30]. Moreover, the IL-6 could downregulate miR-200s, thereby inhibiting PI3K/AKT/GSK pathway activation and glycogen synthesis [31]. Similarly, miR-25, which acts as an antioxidant and as anti-apoptotic by activating the PTEN/Akt pathway, was downregulated in diabetics [15]. Furthermore, the results suggested that lncRNA MEG8 was downregulated in T2DM. Previous studies have also found that MEG8 may upregulate miR-770-5p by decreasing methylation of the miR-770-5p [32]. Nevertheless, the current study did not find differential expressions of miR-770-5p, which requires further exploration.

Bioinformatics analysis showed that DERNAs represent a tight network of many target genes in pathways associated with T2DM. GO function and KEGG pathway analysis suggested that DERNAs were significantly enriched in diabetes-related regulatory processes, including regulation of insulin secretion, carbohydrate metabolism, response to glucose, vitamin metabolism, and lipid metabolism. GO term network showed that 39 mRNAs were anticipated to participate in T2DM, including ACOXL, MGLL, DDB1, PLCB1, GOT1, PRKN, and FUT8. Previous studies found that *DDB1*-mediated degradation of Cry1 was an important target for insulin action on glucose homeostasis, and the *FUT8* was strongly associated with increased susceptibility to diabetes [33,34]. In addition, high glucose stimulation significantly reduced *PRKN* gene expression, and *GOT1* is highly potentially related to glycolipid metabolism [35]. Earlier studies showed that *PLCB1* in pancreatic *β*-cells potentiated G_p_-coupled receptor-dependent insulin secretion. At the same time, the present study also suggested that *PLCB1* was involved in the G_p_- receptors signaling pathway, the metabolic process of phosphatidylinositol, and Wnt signaling pathway [36]. Moreover, consistent with previous findings, we also found that *AGTR2*, *ACOXL*, *MGLL*, *PANK2*, *MID1IP1,* and *LONP2* were associated with lipid catabolism and transport [37,38,39,40]. The PPI network revealed key regulatory genes, including *AGTR2*, *SCD5*, *ST8SIA1*, *GOT1*, *PLCB1,* and *MGLL.* Interestingly, previous studies also found that lipid metabolism and insulin sensitivity were improved in SCD-deficient mice; and *ST8SIA1* was involved in the regulation of insulin secretion [41,42].

Through MREs, lncRNAs, miRNAs, and mRNAs constituted the lncRNA-miRNA-mRNA regulatory network. The increasing studies suggested that the lncRNA-miRNA-mRNA network had an influential role in the physiological regulation of cardiovascular diseases, cancer, as well as other diseases and therefore represented a new therapeutic strategy [43,44]. The current study showed that miR-199b-5p targeted *ATP6V1C2* and *PRKN*. Consistently, in earlier studies, miR-199 negatively affected pancreatic *β*-cell function and was significantly increased in the islets of diabetic mice as well as in the plasma of diabetic [45,46]. This was also confirmed in a subsequent study [47]. This study demonstrated the possible inter-regulatory networks of T2DM-associated miRNAs, mRNAs, and lncRNAs, and further studies on those could reveal critical targets. In summary, aberrant expression of multiple DERNAs and their inter-regulation may affect T2DM pathophysiology. The function of lncRNAs-miRNAs-mRNA regulatory networks in diabetes and related metabolic diseases deserve further investigation.

MiRNAs are present at consistent and reproducible levels in human peripheral blood with high stability and even resistance to RNase activity, suggesting that peripheral miRNAs may be a novel source of highly accessible biomarkers of the disease. And for that, the alteration of miRNAs in the circulation and the construction of the lncRNA-miRNA-mRNA regulatory network through miRNA may provide more accurate information about the pathophysiology of T2DM. Furthermore, the participants were newly diagnosed with T2DM, which enhances the reliability of the findings presented in this study. Several limitations in this study should be noted. First, the sample size was small and human samples may be influenced by various factors, although we tried our best to control them when selecting samples for this study, these traits of the present study may affect the accuracy of the extrapolation of the results. Thus, the next step is to increase the sample size to validate the results. However, this study was a case-control study, which may improve the accuracy. Second, this was only a preliminary exploration that may provide new insights into the molecular mechanisms underlying the development of T2DM, and the results need to be further validated experimentally.

## 5. Conclusions

Through the present case-control study, some new and previously identified lncRNAs, miRNAs, and mRNAs were found significantly differentially expressed in new-onset T2DM. GO function, PPI network, and lncRNA-miRNA-mRNA network identified several DEmRNAs such as SLC25A4, PLCB1, AGTR2, PRKN, and SCD5 were possibly associated with new-onset T2DM, and miR-199b-5p, miR-202-5p, miR-548o-3p as well as miR-1255b-5p could be involved in their regulation. The identification of biomarkers of T2DMs can help in the early detection of patients at risk and the prevention of T2DM. The study of these circulating RNAs altered in T2DM may provide new insights into the identification of specific biomarkers or molecular therapeutic targets, as well as a basis for elucidating their pathogenesis and seeking breakthroughs for further research.

## Figures and Tables

**Table 1:** Descriptive characteristics of participants.

Variables	T2DM (*n* = 24)	NGT (*n* = 24)	*p* Value
Men, n (%)	10 (41.67)	10 (41.67)	1.000
Age (years, mean ± SD)	56.42 ± 8.55	56.46 ± 8.76	0.987
BMI (kg/m^2^, mean ± SD)	26.54 ± 2.56	26.03 ± 2.71	0.507
SBP (mmHg, mean ± SD)	134.89 ± 17.84	126.29 ± 18.49	0.108
DBP (mmHg, mean ± SD)	85.07 ± 9.18	80.04 ± 9.75	0.072
FBG, (mmol/L, mean ± SD)	8.36 ± 1.22	5.36 ± 0.38	<0.001
INS, (mIU/L, mean ± SD)	15.12 ± 13.83	8.67 ± 2.76	0.034

Abbreviations: SD, standard deviation; SBP, systolic blood pressure; BMI, body mass index; DBP, diastolic blood pressure; FBG, fasting blood glucose; T2DM, type 2 diabetes mellitus; INS, insulin; NGT, normal glucose tolerance.

**Table 2:** Enrichment analysis of top 4 MCODE genes function.

MCODE	GO	Description	Log10 (*P*)
MCODE-a	GO:0002016	Regulation of blood volume by renin-angiotensin	−8.74
MCODE-a	GO:0001817	Regulation of cytokine production	−5.39
MCODE-a	GO:0045859	Regulation of protein kinase activity	−2.90
MCODE-a	C0853897	Diabetic Cardiomyopathies	−6.10
MCODE-b	WP1423	Ganglio sphingolipid metabolism	−10.11
MCODE-c	GO:0005975	Carbohydrate metabolic process	−4.44
MCODE-d	GO:0016042	Lipid catabolic process	−10.84
MCODE-d	R-HSA-434316	Fatty Acids bound to GPR40 (FFAR1) regulate insulin secretion	−9.28
MCODE-d	GO:0010817	Regulation of hormone levels	−3.72

Abbreviations: MCODE, molecular complex detection; GO, Gene Ontology; DEGs, differentially expressed genes.

## Data Availability

The datasets produced and/or analyzed in the current study are available upon reasonable request to the corresponding authors.

## Supplementary Materials

The following supporting information can be downloaded at: https://www.mdpi.com/article/10.3390/genes13112073/s1, Figure S1: Correlation analysis of the samples; Figure S2: The expression profiles of lncRNAs; Figure S3: The expression profiles of Genes; Figure S4: Comparison of the expression of 12 miRNAs in NGT and T2DM subjects. MiRNAs, microRNA; NGT, normal glucose tolerance; T2DM, type 2 diabetes mellitus. * *p* < 0.05, ** *p* < 0.01; Figure S5: Comparison of the expression of 12 lncRNAs in NGT and T2DM subjects. LncRNAs, long non-coding RNAs; NGT, normal glucose tolerance; T2DM, type 2 diabetes mellitus. * *p* < 0.05; ** *p* < 0.01; Figure S6: Comparison of the expression of 12 mRNAs in NGT and T2DM subjects. mRNA, messenger RNA; NGT, normal glucose tolerance; T2DM, type 2 diabetes mellitus. * *p* < 0.05; Table S1: Descriptive characteristics of eight mixed samples; Table S2: DEmiRNAs; Table S3: DElncRNAs; Table S4: DEmRNAs; Table S5: Correlation analysis of DEmiRNAs and HOMA-IR. Correlation coefficients were calculated using Spearman correlation analysis. Abbreviations: DEmiRNAs, differentially expressed microRNA; IR, insulin resistance; Table S6: Correlation analysis of DElncRNAs and HOMA-IR. Correlation coefficients were calculated using Spearman correlation analysis. Abbreviations: DElncRNAs, differentially expressed long noncoding RNA; IR, insulin resistance; Table S7: Correlation analysis of DEmRNAs and HOMA-IR. Correlation coefficients were calculated using Spearman correlation analysis. Abbreviations: DEmRNAs, differentially expressed messenger RNA; IR, insulin resistance; Table S8: Functional groups in GO term network analysis; Table S9: Diabetes-related DEGs in GO term network analysis; Table S10: The top 20 core node and their corresponding degree; Table S11: CeRNA connectivity (top 20).
