# Supplementary material for: Differential Expression of lncRNA-miRNA-mRNA and Their Related Functional Networks in New-Onset Type 2 Diabetes Mellitus among Chinese Rural Adults
Source: Genes (Basel). 2022 Nov 9;13(11):2073. doi: 10.3390/genes13112073 (PMC9690016; doi:10.3390/genes13112073)
Supplement: Supplementary file 1 [file genes-13-02073-s001.zip › genes-1908515-supplementary.pdf]

Supplementary Materials

Table S1. Descriptive characteristics of eight mixed samples.

Table S2. DEmiRNAs.

Table S3. DElncRNAs.

Table S4. DEmRNAs.

Table S5. Correlation analysis of DEmiRNAs and HOMA-IR. Correlation coefficients were calculated using Spearman correlation analysis. Abbreviations: DEmiRNAs, differentially expressed microRNA; IR, insulin resistance.

Table S6. Correlation analysis of DElncRNAs and HOMA-IR. Correlation coefficients were calculated using Spearman correlation analysis. Abbreviations: DElncRNAs, differentially expressed long noncoding RNA; IR, insulin resistance.

Table S7. Correlation analysis of DEmRNAs and HOMA-IR. Correlation coefficients were calculated using Spearman correlation analysis. Abbreviations: DEmRNAs, differentially expressed messenger RNA; IR, insulin resistance.

Table S8. Functional groups in GO term network analysis.

Table S9. Diabetes-related DEGs in GO term network analysis.

Table S10. The top 20 core node and their corresponding degree.

Table S11. CeRNA connectivity (top 20).

Figure S1. Correlation analysis of the samples.

Figure S2. The expression profiles of lncRNAs.

Figure S3. The expression profiles of Genes.

Figure S4. Comparison of the expression of 12 miRNAs in NGT and T2DM subjects. MiRNAs, microRNA; NGT, normal glucose tolerance; T2DM, type 2 diabetes mellitus. $^{*}p$ <0.05, $^{**}p$ <0.01.

Figure S5. Comparison of the expression of 12 lncRNAs in NGT and T2DM subjects. LncRNAs, long non-coding RNAs; NGT, normal glucose tolerance; T2DM, type 2 diabetes mellitus. $^{*}p$ <0.05; $^{**}p$ <0.01.

Figure S6. Comparison of the expression of 12 mRNAs in NGT and T2DM subjects. mRNA, messenger RNA; NGT, normal glucose tolerance; T2DM, type 2 diabetes mellitus. $^{*}p$ <0.05.

Table S1. Descriptive characteristics of eight mixed samples

Variables	Case 1	Case 2	Case 3	Case 4	Control 1	Control 2	Control 3	Control 4
Men, n (%)	2 (33.33)	2 (33.33)	3 (50.00)	3 (50.00)	2 (33.33)	2 (33.33)	3 (50.00)	3 (50.00)
Age (years, mean ± SD)	54.83 ± 9.00	56.50 ± 8.76	57.00 ± 8.74	57.33 ± 9.87	54.66 ± 9.31	56.17 ± 8.89	57.33 ± 9.00	57.67 ± 9.99
BMI (kg/m^2, mean ± SD)	26.55 ± 2.25	25.55 ± 2.29	28.19 ± 3.42	25.89 ± 1.80	25.78 ± 2.43	26.18 ± 3.71	26.00 ± 2.47	26.18 ± 2.80
SBP (mmHg, mean ± SD)	131.94 ± 17.79	130.28 ± 15.37	131.33 ± 11.59	146.00 ± 24.04	116.78 ±14.84	128.44 ±14.49	136.28 ± 24.95	123.67 ± 16.64
DBP (mmHg, mean ± SD)	83.00 ± 8.80	80.67 ± 4.52	84.50 ± 9.57	92.11 ± 10.48	76.28 ± 6.81	82.22 ± 10.44	83.72 ± 12.10	77.94 ± 9.55
FBG, (mmol/L, mean ± SD)	7.72 ± 0.56	8.28 ± 0.76	9.37 ± 1.78	8.08 ± 0.99	5.42 ± 0.60	5.23 ± 0.41	5.45 ± 0.19	5.35 ± 0.24
INS, (mIU/L, mean ± SD)	10.43 ± 3.22	12.48 ± 3.13	23.98 ± 26.66	13.60 ± 4.08	8.77 ± 2.21	9.24 ± 3.28	9.96 ± 3.24	6.71 ± 1.38
HOMA-IR, (mean ± SD)	3.61 ± 1.26	4.59 ± 1.25	10.14 ± 10.99	4.89 ± 1.47	2.14 ± 0.72	2.19 ± 0.92	2.42 ± 0.81	1.61 ± 0.41

Abbreviations: BMI, body mass index; SBP, systolic blood pressure; DBP, diastolic blood pressure; FBG, fasting blood glucose; INS, insulin.

Table S2. DEmiRNAs

ID	*p* value	Log_2 (fold change)	target genes_Num	terms
miR-199b-5p	0.044	11.606	6	GO:0006629: lipid metabolic process; GO:0005509: calcium ion binding.
miR-190	0.021	11.032		
miR-484	0.014	6.863	9	GO:0009743: response to carbohydrate; GO:0001523: retinoid metabolic process; GO:0006629: lipid metabolic process; GO:0009749: response to glucose; GO:1900077: negative regulation of cellular response to insulin stimulus.
miR-3944-5p	0.044	5.028	8	GO:0009749: response to glucose; GO:0030073: insulin secretion; GO:0032024: positive regulation of insulin secretion.
miR-200c-3p	0.029	-2.735	15	GO:0000165: MAPK cascade; GO:0007165: signal transduction; GO:0043087: regulation of GTPase activity;
miR-871	0.000	-3.724	6	GO:0043488: regulation of mRNA stability; GO:0000187: activation of MAPK activity.
miR-50	0.000	-5.220	5	GO:0006796: phosphate-containing compound metabolic process; GO:0006811: ion transport;
miR-351	0.003	-7.129	7	GO:0000165: MAPK cascade; GO:0007165: signal transduction.
miR-574	0.048	-9.932		
miR-5129	0.021	-10.538		
miR-25-5p	0.026	-11.012	3	GO:0000187: activation of MAPK activity; GO:0001666: response to hypoxia; GO:0001934: positive regulation of protein phosphorylation.
miR-1255b-5p	0.008	-11.168	11	GO:0006796: phosphate-containing compound metabolic process; GO:0006811: ion transport; GO:0006814: sodium ion transport;

Table S3. DElncRNAs

ID	Symbol	*p* value	Log_2 (fold change)
ENST00000379053	LINC00654	0.019	2.559
ENST00000524745	ZBED9	0.013	-2.675
ENST00000527986	WAC-AS1	0.020	-4.359
ENST00000562900	AC009035.1	0.014	-1.973
ENST00000566814	Z95115.1	0.020	-3.645
ENST00000582452	LINC00683	0.002	-1.204
ENST00000636531	AC006453.3	0.023	-5.352
ENST00000650382	AL031315.1	0.010	3.485
ENST00000651360	PRR26	0.021	-2.874
ENST00000659693	LINC01361	0.003	4.052
ENST00000664311	LINC02798	0.016	3.467
ENST00000666972	MEG8	0.008	-1.692

Table S4. DEmRNAs

ID	Symbol	Log$_2$ (fold change)	terms
ENSG00000110013	*SIAE*	-1.741	GO:0002682: regulation of immune system process; GO:0005975: carbohydrate metabolic process.
ENSG00000163281	*GNPDA2*	2.428	GO:0005975: carbohydrate metabolic process; GO:0006043 glucosamine catabolic process.
ENSG00000198730	*CTR9*	-2.385	GO:0000993: RNA polymerase II core binding; GO:0016055: Wnt signaling pathway.
ENSG00000186314	*PRELID2*	-2.482	GO:0015914: phospholipid transport.
ENSG00000256660	*CLEC12B*	-2.720	GO:0019903: protein phosphatase binding; GO:0030246: carbohydrate binding.
ENSG00000204406	*MBD5*	0.622	GO:0042593: glucose homeostasis; GO:0060399: positive regulation of growth hormone receptor signaling pathway.
ENSG00000177000	*MTHFR*	-1.393	Ko01100: Metabolic pathways; ko01200: Carbon metabolism; ko01523: Antifolate resistance; ko00670: One carbon pool by folate.
ENSG00000120053	*GOT1*	-3.388	GO:0006094: gluconeogenesis; GO:0006114: glycerol biosynthetic process; GO:0032869: cellular response to insulin stimulus.
ENSG00000059804	*SLC2A3*	-3.217	GO:0005355: glucose transmembrane transporter activity; GO:0005536: glucose binding.
ENSG00000143882	*ATP6V1C2*	-1.885	GO:0008286: insulin receptor signaling pathway; GO:0030177: positive regulation of Wnt signaling pathway.
ENSG00000169100	*SLC25A6*	2.019	GO:0050796: regulation of insulin secretion; GO:0005471: ATP, ADP antiporter activity.
ENSG00000151729	*SLC25A4*	-2.852	GO:0006626: protein targeting to mitochondrion; GO:0050796: regulation of insulin secretion.
ENSG00000153093	*ACOXL*	-1.181	GO:0006631; fatty acid metabolic process;GO:0006635; fatty acid beta-oxidation;GO:0008150; biological_process;GO:0033540; fatty acid beta-oxidation using acyl-CoA oxidase;GO:0055088; lipid homeostasis;GO:0055114; oxidation-reduction process.
ENSG00000166106	*ADAMTS15*	3.205	GO:0006508; proteolysis.
ENSG00000180772	*AGTR2*	3.114	GO:0001991; regulation of systemic arterial blood pressure by circulatory renin-angiotensin;GO:0002018; renin-angiotensin regulation of aldosterone production.
ENSG00000183020	*AP2A2*	-2.566	GO:0008289; lipid binding.
ENSG00000129691	*ASH2L*	-4.387	GO:0043627; response to estrogen.
ENSG00000139178	*C1RL*	-1.786	GO:0002376; immune system process.
ENSG00000152495	*CAMK4*	1.438	GO:0002250; adaptive immune response;GO:0002376; immune system process.
ENSG00000154080	*CHST9*	1.480	GO:0005975; carbohydrate metabolic process.

ENSG00000137869	*CYP19A1*	2.087	GO:0002677; negative regulation of chronic inflammatory response;GO:0006694; steroid biosynthetic process;GO:0006703; estrogen biosynthetic process;GO:0006710; androgen catabolic process;GO:0008209; androgen metabolic process.
ENSG00000104325	*DECR1*	-2.082	GO:0006629; lipid metabolic process;GO:0006631; fatty acid metabolic process;GO:0006635; fatty acid beta-oxidation;GO:0055114; oxidation-reduction process.
ENSG00000137942	*FNBP1L*	-1.715	GO:0005515; protein binding;GO:0008289; lipid binding;GO:0045296; cadherin binding;GO:0051020; GTPase binding.
ENSG00000033170	*FUT8*	-1.921	GO:0007179; transforming growth factor beta receptor signaling pathway;GO:0007229; integrin-mediated signaling pathway.
ENSG00000175229	*GAL3ST3*	6.741	GO:0005996; monosaccharide metabolic process; GO:0009247; glycolipid biosynthetic process.
ENSG00000133895	*MEN1*	2.129	GO:0071333; cellular response to glucose stimulus;GO:0071375; cellular response to peptide hormone stimulus.
ENSG00000008130	*NADK*	7.704	GO:0035774; positive regulation of insulin secretion involved in cellular response to glucose stimulus;GO:0046034; ATP metabolic process.
ENSG00000125779	*PANK2*	1.254	GO:0090207; regulation of triglyceride metabolic process;GO:1904251; regulation of bile acid metabolic process
ENSG00000101333	*PLCB4*	2.338	GO:0006629; lipid metabolic process.
ENSG00000154822	*PLCL2*	1.642	GO:0016042; lipid catabolic process.
ENSG00000179598	*PLD6*	7.036	GO:0006629; lipid metabolic process;GO:0006654; phosphatidic acid biosynthetic process.
ENSG00000185345	*PRKN*	-3.353	GO:0032368; regulation of lipid transport;GO:0046676; negative regulation of insulin secretion.
ENSG00000186350	*RXRA*	2.670	GO:0003707; steroid hormone receptor activity.
ENSG00000146411	*SLC2A12*	3.019	GO:0008643; carbohydrate transport;GO:0008645; hexose transport;GO:0046323; glucose import;GO:0055085; transmembrane transport;GO:1902600; hydrogen ion transmembrane transport;GO:1904659; glucose transmembrane transport
ENSG00000022567	*SLC45A4*	4.637	O:0015770; sucrose transport.
ENSG00000065609	*SNAP91*	-3.121	GO:0000149; SNARE binding;GO:0005515; protein binding;GO:0005543; phospholipid binding.
ENSG00000111728	*ST8SIA1*	0.792	GO:0005975; carbohydrate metabolic process;GO:0006486; protein glycosylation;GO:0006629; lipid metabolic process.
ENSG00000101849	*TBL1X*	2.100	GO:0019216; regulation of lipid metabolic process.

Table S5. Correlation analysis of DEmiRNAs and HOMA-IR.

ID	Correlation coefficient[a]	*p* value
miR-199b-5p	0.445	0.317
miR-190	0.964	<0.001
miR-484	0.613	0.144
miR-3944-5p	0.482	0.274
miR-200c-3p	0.214	0.645
miR-871	-0.929	0.003
miR-50	-0.893	0.007
miR-351	-0.741	0.057
miR-574	-0.906	0.005
miR-5129	-0.906	0.005
miR-25-5p	-0.445	0.317
miR-1255b-5p	-0.867	0.012

[a]Correlation coefficients were calculated using Spearman correlation analysis. Abbreviations: DEmiRNAs, differentially expressed microRNA; IR, insulin resistance.

Table S6. Correlation analysis of DElncRNAs and HOMA-IR.

ID	Symbol	Correlation coefficient[a]	*p* value
ENST00000379053	LINC00654	0.607	0.148
ENST00000524745	ZBED9	-0.607	0.148
ENST00000527986	WAC-AS1	-0.741	0.057
ENST00000562900	AC009035.1	-0.750	0.052
ENST00000566814	Z95115.1	-0.775	0.041
ENST00000582452	LINC00683	-0.500	0.253
ENST00000636531	AC006453.3	-0.704	0.077
ENST00000650382	AL031315.1	0.631	0.129
ENST00000651360	PRR26	-0.964	<0.001
ENST00000659693	LINC01361	0.523	0.229
ENST00000664311	LINC02798	0.649	0.115
ENST00000666972	MEG8	-0.714	0.071

[a]Correlation coefficients were calculated using Spearman correlation analysis. Abbreviations: DElncRNAs, differentially expressed long noncoding RNA; IR, insulin resistance.

Table S7. Correlation analysis of DEmRNAs and HOMA-IR.

ID	Symbol	Correlation coefficient[a]	*p* value
ENSG00000110013	*SIAE*	-0.857	0.014
ENSG00000163281	*GNPDA2*	0.750	0.052
ENSG00000198730	*CTR9*	-0.286	0.535
ENSG00000186314	*PRELID2*	-0.750	0.052
ENSG00000256660	*CLEC12B*	-0.685	0.090
ENSG00000204406	*MBD5*	0.429	0.337
ENSG00000177000	*MTHFR*	-0.571	0.180
ENSG00000120053	*GOT1*	-0.964	<0.001
ENSG00000059804	*SLC2A3*	-0.857	0.014
ENSG00000143882	*ATP6V1C2*	-0.679	0.094
ENSG00000169100	*SLC25A6*	0.468	0.289
ENSG00000151729	*SLC25A4*	-0.857	0.014

[a]Correlation coefficients were calculated using Spearman correlation analysis. Abbreviations: DEmRNAs, differentially expressed messenger RNA; IR, insulin resistance.

Table S8. Functional groups in GO term network analysis.

GOfunctional groups	The most significant functional	Corrected *p* value[a]
Group-1	Trans-synaptic signaling by endocannabinoid	0.00005802
Group-2	Interieukin-1 beta production	0.00230861
Group-3	Regulation of establishment of endothelial barrier	0.00217613
Group-4	Fusion of virus membrance with host plasma membrane	0.00004160
Group-5	Regulation of lipase activity	0.00000665
Group-6	Postsynaptic signal transduction	0.00000001
Group-7	Adenylate cyclase-modulating G protein-coupled receptor signaling pathway	0.00000007
Group-8	Tricarboxylic acid cycle	0.00004416
Group-9	Wnt signaling pathway calcium modulating pathway	<0.00000001
Group-10	Polyol metabolic process	<0.00000001
Group-11	Phospholipase C-activating G protein-coupled receptor signaling pathway	0.00000002
Group-12	Mitochondrial ATP synthesis coupled proton transport	<0.00000001
Group-13	Negative regulation of cell growth involved in cardiac muscle cell development	<0.00000001
Group-14	Lipid catabolic process	<0.00000001
Group-15	Glycosphingolipid biosynthetic process	0.00090846

[a]Adjusted group *p* value corrected with Bonferroni step down procedure. Abbreviations: GO, Gene Ontology.

Table S9. Diabetes-related DEGs in GO term network analysis

GO Term ID	GO functional groups	GO Term	Corrected *p* value[a]	Associated DEGs
GO:1905953	Group-13, 14	Negative regulation of lipid localization	0.0025	*AGTR2*
GO:1905952	Group-5, 14	Regulation of lipid localization	0.0039	*CYP19A1, PRKN, RNRA*
GO:1903522	Group-11, 13	Regulation of blood circulation	0.0004	*DLG1*
GO:1901137	Group-12	Carbohydrate derivative biosynthetic process	<0.0001	*ALG1, ATP5F1E, CEMIP, CHST9, DCTD, FUT8, GAL3ST3*
GO:0090207	Group-14	Regulation of triglyceride metabolic process	0.0010	*PANK2*
GO:0060191	Group-5, 14	Regulation of lipase activity	0.0001	*ARL1, BICD1*
GO:0046889	Group-14	Positive regulation of lipid biosynthetic process	0.0139	*MID1IP1, WNT4,*
GO:0045923	Group-14	Positive regulation of fatty acid metabolic process	0.0139	*MID1IP1*
GO:0045913	Group-10, 11, 13, 14	Positive regulation of carbohydrate metabolic process	0.0111	*DDB1*
GO:0045834	Group-5, 14	Positive regulation of lipid metabolic process	0.0005	*MID1IP1, WNT4*
GO:0045744	Group-13	Negative regulation of G protein-coupled receptor signaling pathway	0.0056	*BICD1, LY6G6E*
GO:0044242	Group-14	Cellular lipid catabolic process	0.0008	*ACOXL, DECR1, LONP2, MGLL, SPHK1*
GO:0043648	Group-8	Dicarboxylic acid metabolic process	0.0046	*ATCAY, GOT1, MTHFR, PCDHGA5*
GO:0043647	Group-10	Inositol phosphate metabolic process	0.0001	*PLCB1, PLCB4*
GO:0043470	Group-14	Regulation of carbohydrate catabolic process	0.0129	*POM121,*
GO:0032651	Group-2	Regulation of interleukin-1 beta production	0.0115	*SPHK1*
GO:0016042	Group-14	Lipid catabolic process	<0.0001	*ACOXL, CYP19A1, DECR1, LONP2, MGLL, PLCB1, PLCB4, PLCL2, PLD6, SPHK1*
GO:0015908	Group-13, 14	Fatty acid transport	0.0122	*AGTR2, MID1IP1*
GO:0010675	Group-10, 14	Regulation of cellular carbohydrate metabolic process	0.0141	*DDB1, PRKN*
GO:0010517	Group-5	Regulation of phospholipase activity	0.0016	*BICD1, ARL1*
GO:0008277	Group-6, 13	Regulation of G protein-coupled receptor signaling pathway	0.0141	*BICD1, LY6G6E, MGLL, PLCB1, RGS1*
GO:0007223	Group-6, 9, 10	Wnt signaling pathway, calcium modulating pathway	0.0001	*PLCB1,*
GO:0007213	Group-6, 10, 13	G protein-coupled acetylcholine receptor signaling pathway	0.0001	*PLCB1*
GO:0007200	Group-11, 14	Phospholipase C-activating G protein-coupled receptor signaling pathway	<0.0001	*BICD1*
GO:0007193	Group-7, 9, 13	Adenylate cyclase-inhibiting G protein-coupled receptor signaling pathway	0.0022	*RGS1*
GO:0007188	Group-7, 9	Adenylate cyclase-modulating G protein-coupled receptor signaling pathway	<0.0001	*ADGRB3, PTH2R, RGS1*
GO:0006688	Group-15	Glycosphingolipid biosynthetic process	0.0076	*ST8SIA1*

GO:0006641	Group-14	Triglyceride metabolic process	0.0008	*MGLL*, *PANK2*,
GO:0006635	Group-14	Fatty acid beta-oxidation	0.0111	*ACOXL*, *DECR1*, *LONP2*
GO:0006107	Group-8	Oxaloacetate metabolic process	0.0007	*GOT1*,
GO:0006099	Group-8	Tricarboxylic acid cycle	0.0001	*PCDHGA5*,
GO:0006094	Group-8	Gluconeogenesis	0.0114	*DDB1*, *GOT1*
GO:0003018	Group-11, 13, 14	Vascular process in circulatory system	0.0002	*AGTR2*, *SLC2A3*

[a]Adjusted group *p* value corrected with Bonferroni step down procedure. Abbreviations: Associated DEGs, differentially expressed genes associated with the regulation of diabetes; GO, Gene Ontology.

Table S10. The top 20 core nodes and their corresponding degree.

Node	Degree	Domain summary
GNAQ	14	Guanine nucleotide-binding protein G(q) subunit alpha
AGT	11	Angiotensinogen; Essential component of the renin-angiotensin system
CCR5	11	C-C chemokine receptor type 5
CXCR4	11	C-X-C chemokine receptor type 4
ADRBK1	9	Beta-adrenergic receptor kinase 1
AGTR2	9	Type-2 angiotensin II receptor
CD4	9	T-cell surface glycoprotein CD4
PLCB1	8	1-phosphatidylinositol 4,5-bisphosphate phosphodiesterase beta-1
AGTR1	7	Type-1 angiotensin II receptor
GRM5	6	Metabotropic glutamate receptor 5; G-protein coupled receptor for glutamate
ITIH4	6	Inter-alpha-trypsin inhibitor heavy chain family member 4
REN	6	Renin
ACE	5	Angiotensin-converting enzyme
CD28	5	T-cell-specific surface glycoprotein CD28
GLUD1	5	Glutamate dehydrogenase 1, mitochondrial
GPT2	5	Alanine aminotransferase 2
LCK	5	Tyrosine-protein kinase Lck
LIPE	5	Hormone-sensitive lipase
MGLL	5	Monoglyceride lipase
RGS4	5	Regulator of G-protein signaling 4

Table S11. CeRNA connectivity (top 20).

ID	Type	Degree	up_down
MiR-214-3p	miRNA	14	down
MiR-484	miRNA	13	up
MiR-1255b-5p	miRNA	11	down
MiR-548o-3p	miRNA	10	up
MiR-199b-5p	miRNA	9	up
MiR-8	miRNA	8	up
MiR-371a-5p	miRNA	6	down
MiR-4433b-5p	miRNA	6	down
MiR-372-3p	miRNA	5	down
MiR-25-5p	miRNA	5	down
ADAMTS15	mRNA	4	up
MiR-351-3p	miRNA	4	down
MiR-3944-5p	miRNA	4	up
ENST00000664283	lncRNA	4	down
PRKN	mRNA	3	down
CAMK4	mRNA	3	up
ENST00000379053	lncRNA	3	up
CYP19A1	mRNA	3	up
ENST00000659693	lncRNA	3	up
MiR-202-5p	miRNA	3	down

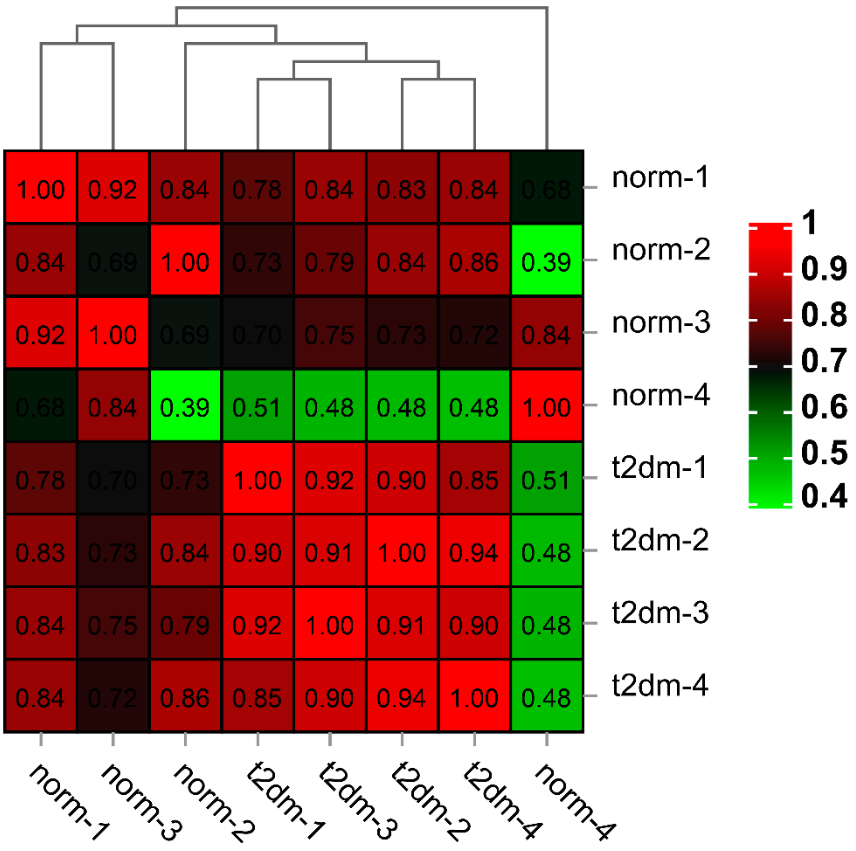

Figure S1. Samples correlation analysis

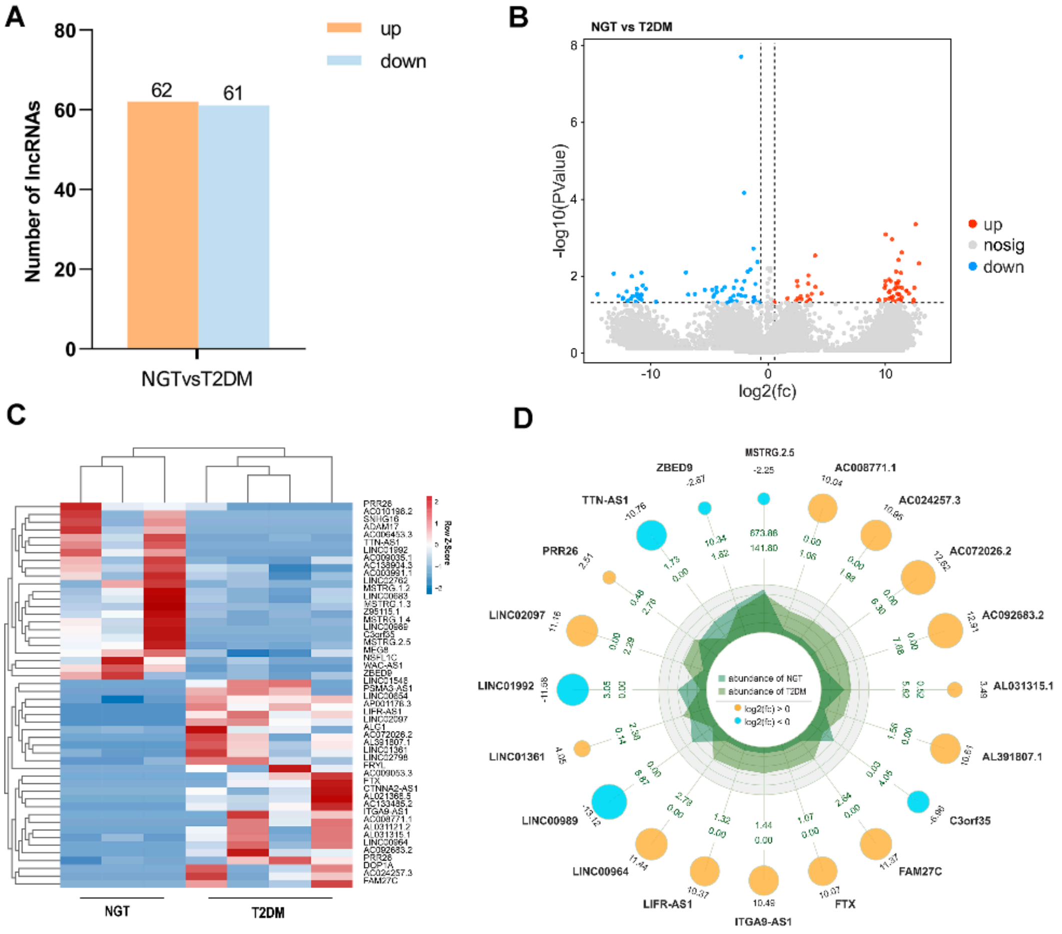

Figure S2. The expression profiles of lncRNA. (A) The column chart of the number of statistical DElncRNA. (B) The volcano plot displays the distribution of DElncRNA. (C) Hierarchical clustering analysis of differentially expressed lncRNAs between T2DM group and NGT group were showed by the heat map. Red colour represents up-regulated lncRNAs, and the blue colour represents down-regulated lncRNAs. (D) The radar plots display the distribution of DElncRNA and the size of the circle indicates the log_2 (FC). LncRNA, long noncoding RNA; NGT, normal glucose tolerance; T2DM, type 2 diabetes mellitus; Log2 (FC), log2(fold change).

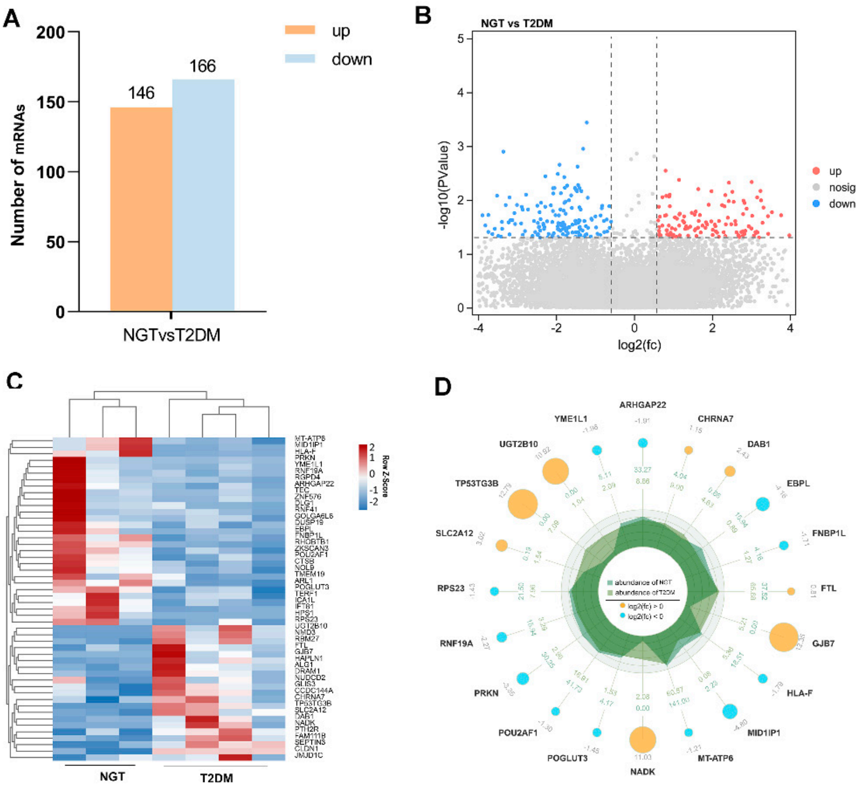

Figure S3. The expression profiles of Genes. (A) The column chart of the number of statistical DEGs. (B) The volcano plot displays the distribution of DEGs. (C) Hierarchical clustering analysis of differentially expressed mRNAs between T2DM group and NGT group were showed by the heat map. Red colour represents up-regulated mRNAs, and the blue colour represents down-regulated mRNAs. (D) The radar plots display the distribution of DEGs and the size of the circle indicates the log2 (FC). DEGs, differentially expressed genes; NGT, normal glucose tolerance; T2DM, type 2 diabetes mellitus; Log2 (FC), log2(fold change).

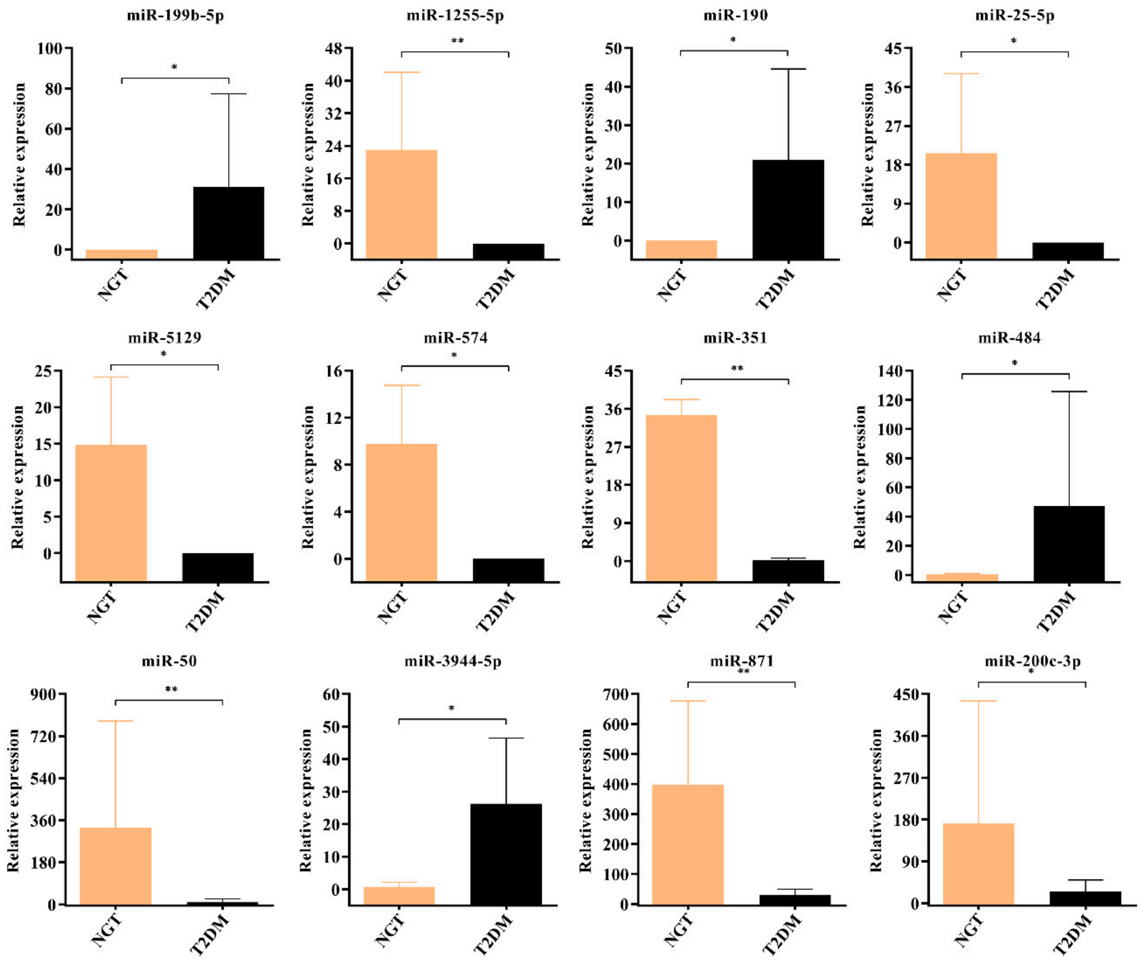

Figure S4. Comparison of the expression of 12 miRNAs in control and T2DM subjects. Column and error bar indicated means and standard deviations. MiRNAs, microRNA; NGT, normal glucose tolerance; T2DM, type 2 diabetes mellitus. $^{*}p$ <0.05, $^{**}p$ <0.01. The *p*-values were obtained using edgeR method.

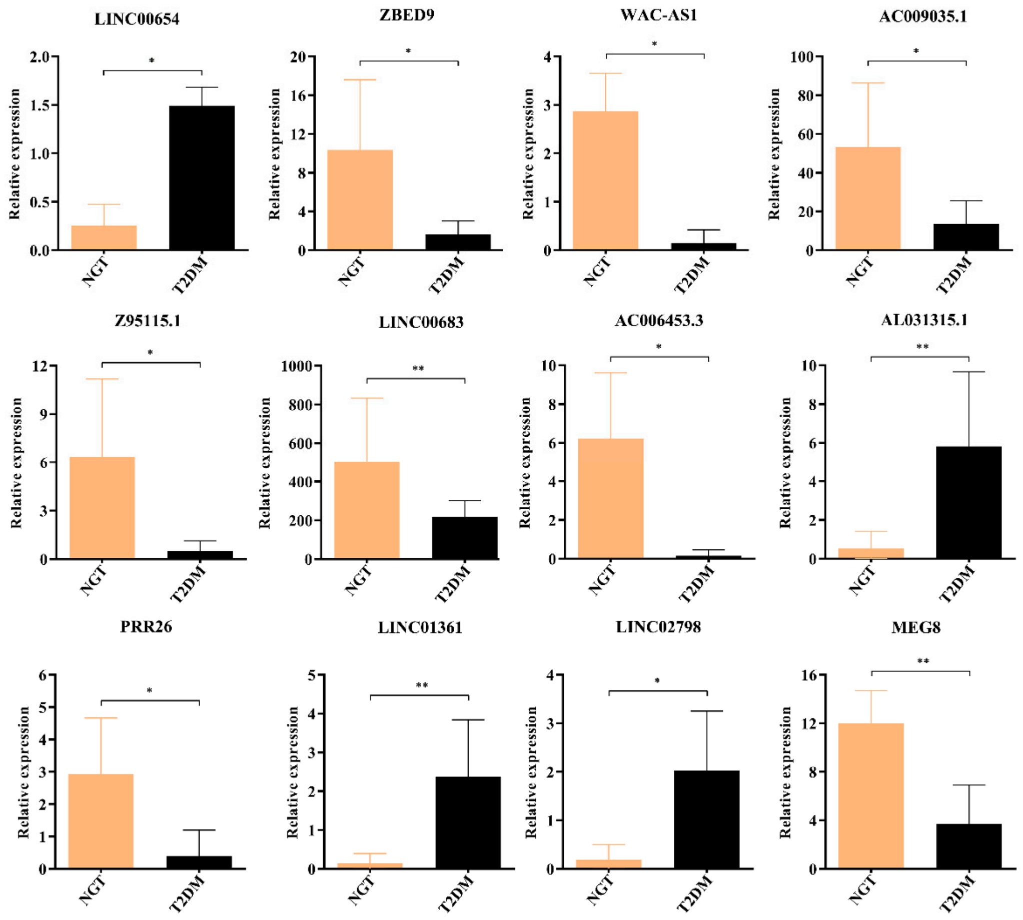

Figure S5. Comparison of the expression of 12 lncRNAs in control and T2DM subjects. Column and error bar indicated means and standard deviations. LncRNAs, long non-coding RNAs; NGT, normal glucose tolerance; T2DM, type 2 diabetes mellitus. $^{*}p < 0.05$; $^{**}p < 0.01$. The p-values were obtained using edgeR method.

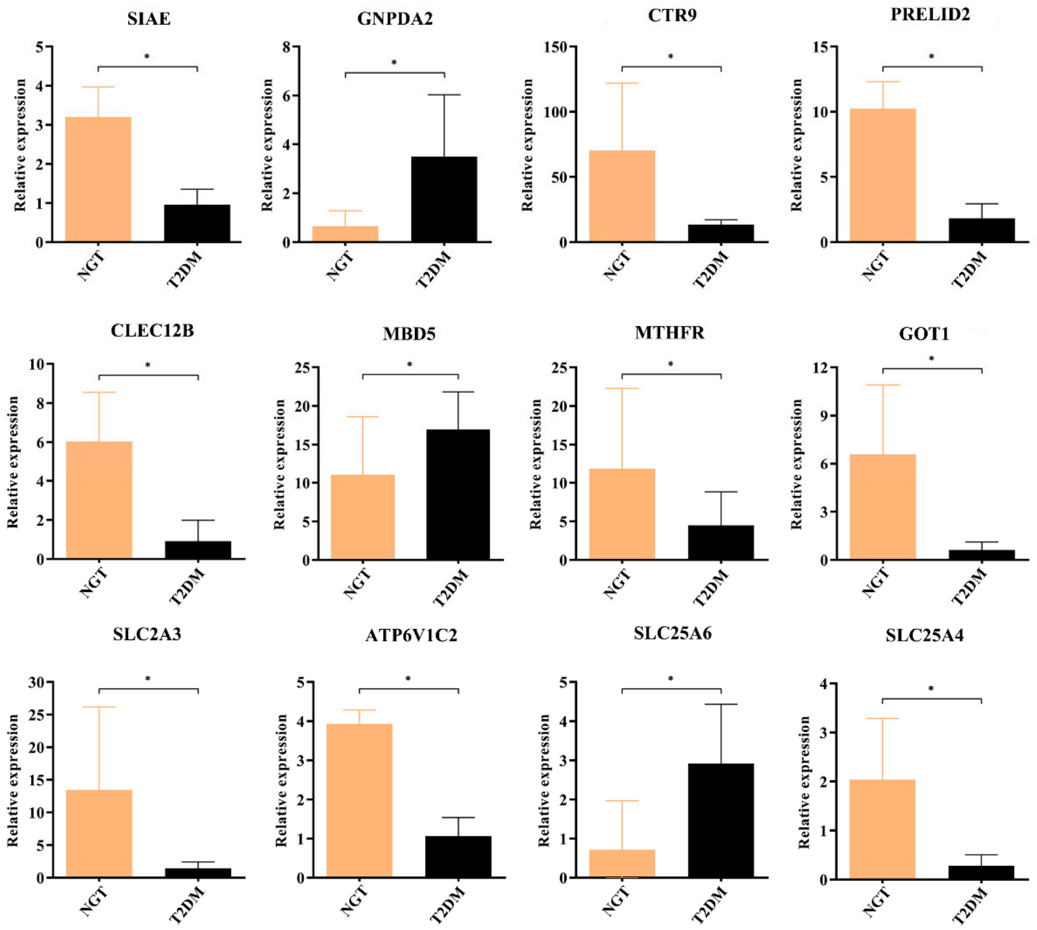

Figure S6. Comparison of the expression of 12 mRNAs in control and T2DM subjects. Column and error bar indicated means and standard deviations. mRNA, messenger RNA; NGT, normal glucose tolerance; T2DM, type 2 diabetes mellitus. $^{*}p$ <0.05; The *p*-values were obtained using edgeR method.
